# Ca^2+^ Microdomains in T-Lymphocytes

**DOI:** 10.3389/fonc.2017.00073

**Published:** 2017-05-02

**Authors:** Insa M. A. Wolf, Andreas H. Guse

**Affiliations:** ^1^The Calcium Signalling Group, Department of Biochemistry and Molecular Cell Biology, University Medical Centre Hamburg-Eppendorf, Hamburg, Germany

**Keywords:** nicotinic acid adenine dinucleotide phosphate, T cell, signal transduction, local Ca^2+^ signals, ryanodine receptors

## Abstract

Early Ca^2+^ signaling is characterized by occurrence of Ca^2+^ microdomains formed by opening of single or clusters of Ca^2+^ channels, thereby initiating first signaling and subsequently activating global Ca^2+^ signaling mechanisms. However, only few data are available focusing on the first seconds and minutes of Ca^2+^ microdomain formation and related signaling pathways in activated T-lymphocytes. In this review, we condense current knowledge on Ca^2+^ microdomain formation in T-lymphocytes and early Ca^2+^ signaling, function of Ca^2+^ microdomains, and microdomain organization. Interestingly, considering the first seconds of T cell activation, a triphasic Ca^2+^ signal is becoming apparent: (i) initial Ca^2+^ microdomains occurring in the first second of T cell activation, (ii) amplification of Ca^2+^ microdomains by recruitment of further channels in the next 5–10 s, and (iii) a transition to global Ca^2+^ increase. Apparently, the second messenger nicotinic acid adenine dinucleotide phosphate is the first second messenger involved in initiation of Ca^2+^ microdomains. Ryanodine receptors type 1 act as initial Ca^2+^ release channels in CD4^+^ T-lymphocytes. Regarding the temporal correlation of Ca^2+^ microdomains with other molecular events of T cell activation, T cell receptor-dependent microdomain organization of signaling molecules Grb2 and Src homology [SH2] domain-containing leukocyte protein of 65 kDa was observed within the first 20 s. In addition, fast cytoskeletal changes are initiated. Furthermore, the involvement of additional Ca^2+^ channels and organelles, such as the Ca^2+^ buffering mitochondria, is discussed. Future research developments will comprise analysis of the causal relation between these temporally coordinated signaling events. Taken together, high-resolution Ca^2+^ imaging techniques applied to T cell activation in the past years paved the way to detailed molecular understanding of initial Ca^2+^ signaling mechanisms in non-excitable cells.

## Introduction

Ca^2+^ signaling modulates a large variety of intracellular downstream targets. How Ca^2+^ signals are converted into meaningful cell responses has been a major area of interest in the past years (1). The specific signature of Ca^2+^ signals in time and space, in other words the spatiotemporal composition of such signals, appears to be very important in that sense. In general, we differentiate between local and global Ca^2+^ signals. Furthermore, differences in the temporal dimension result in transient, oscillatory, or sustained Ca^2+^ signals. Of specific interest for this review will be small local, and usually transient or oscillatory Ca^2+^ signals, also termed Ca^2+^ microdomains.

A sustained global increase of the free cytosolic Ca^2+^ concentration ([Ca^2+^]_i_) is essential for activation of T-lymphocytes, initiating transcriptional regulation, proliferation, and differentiation into effector T-lymphocytes. Activation is accompanied by a decrease in T-lymphocyte motility as well as to rounding up of the cell, thereby enabling immune synapse formation. A major regulator for transcriptional regulation, proliferation, and differentiation is Ca^2+^-dependent activation of calcineurin, and dephosphorylation of nuclear factor of activated T cells (NFAT) and its transport into the nucleus, where NFAT acts as one of the main transcription factors of T cells [reviewed in Ref (2).]. Similarly, amplitude and frequency of Ca^2+^ oscillations in T-lymphocytes are critical for the downstream effects. Ca^2+^ oscillations may enhance the efficiency of signaling to the nucleus (3) and may mediate mitochondrial bioenergetics *via* IP_3_R signaling along the endoplasmic reticulum (ER)–mitochondrial interface [reviewed in Ref (4).]. By contrast, an excessive increase in [Ca^2+^]_i_ drives T cell apoptosis [reviewed in Ref (4).].

Far less understood are origin and impact of Ca^2+^ microdomains in T-lymphocytes, which eventually initiate a sustained increase of [Ca^2+^]_i_ or Ca^2+^ oscillations. Whereas there are plenty of data on Ca^2+^ microdomains, e.g., in cardiomyocytes [reviewed in Ref (5).], there is no systematic review for Ca^2+^ microdomains in immune cells or T-lymphocytes in particular. Therefore, in this review, Ca^2+^ microdomain formation and the underlying putative mechanisms, e.g., ion channels, ion pumps, second messengers, and other factors will be discussed.

## Ca^2+^ Microdomains and Ca^2+^ Signaling

Ca^2+^ microdomains occur due to opening of single Ca^2+^ channels or small clusters of single Ca^2+^ channels, leading to a spatially restricted, usually small increase of [Ca^2+^]_i_. Topological sites for Ca^2+^ microdomains are the surfaces of membranes in the vicinity of Ca^2+^ channels. These membranes may be membranes of Ca^2+^ stores, such as the ER, or the plasma membrane. Already in 1992, it has been suggested that in presynaptic terminals, Ca^2+^ microdomains close to the plasma membrane are crucial for the rapid release of neurotransmitters in the neuronal cleft (6). The ER is a very complicated membranous network, which depending on its specific protein composition may be more tubular or exists rather in form of sheets [reviewed in Ref (7).]. Furthermore, ER associates and moves along with established microtubules in order to create cellular microdomains. As such, the tubular structure and tip attachment complexes of the smooth ER allow for spatially restricted Ca^2+^ signaling domains (7).

Ca^2+^ spreading throughout the cell is limited due to various factors, e.g., sequestration by Ca^2+^-binding proteins or organelles such as mitochondria. Thus, the distance in which Ca^2+^ ions effectively move and may regulate target proteins is around 200–300 nm within the cytosol (8). A more extended increase of [Ca^2+^]_i_, both in time and space, is therefore dependent on second messengers diffusing through the cell and on recruitment of multiple Ca^2+^ release and/or entry channels.

The underlying mechanisms of microdomain organization are at least (a) formation, metabolism, diffusion, and buffering of Ca^2+^-mobilizing second messengers, (b) Ca^2+^ channel activation and recruitment, (c) Ca^2+^ pump activation and recruitment, and (d) distribution of Ca^2+^ buffers.

**Figure 1:**
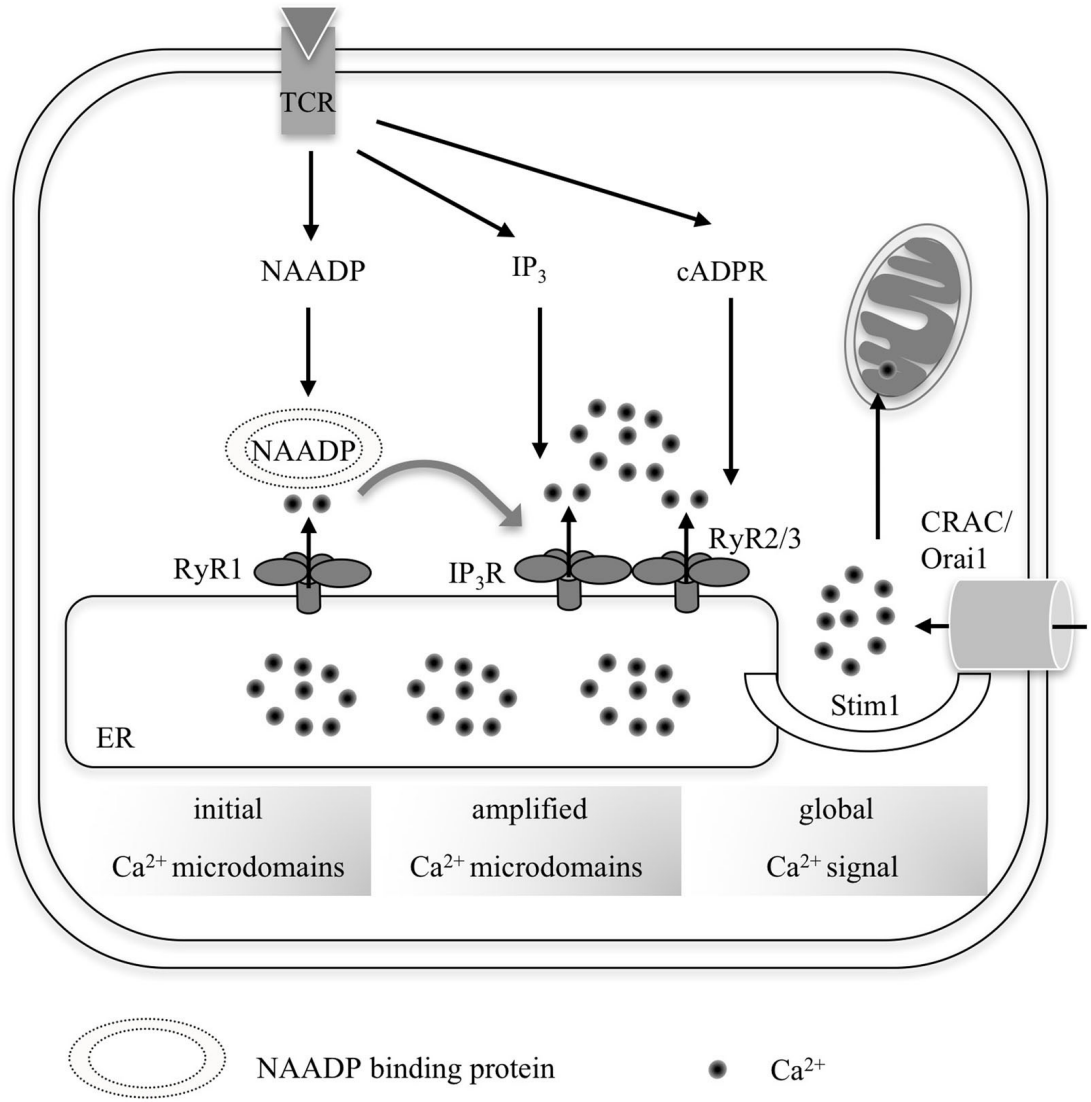
**Overall scheme summarizing the progression from local Ca^2+^ microdomains to a global increase of [Ca^2+^] in (CD4^+^) T-lymphocytes**. Upon TCR activation by its respective cognate antigenic peptide, three second messengers are formed consecutively: nicotinic acid adenine dinucleotide phosphate (NAADP), IP_3_, and cyclic ADP-ribose (cADPR). NAADP presumably binds to an accessory binding protein before targeting RyR1, which generates early Ca^2+^ microdomains during the first seconds of T cell activation. This so-called trigger Ca^2+^ then facilitates activation of further channels such as IP_3_R and RyR2/3 in concert with the respective second messengers IP_3_ and cADPR during the pacemaker phase of Ca^2+^ activation (3–10 min upon activation). Ca^2+^ depletion of the endoplasmic reticulum (ER) leads to Ca^2+^ release-activated Ca^2+^ entry *via* Stim1 and Orai1 during global Ca^2+^ signaling.

(a) Ca^2**+**^-mobilizing second messengers: Ca^2+^-mobilizing second messengers, such as d-*myo*-inositol 1,4,5-trisphosphate (IP_3_), nicotinic acid adenine dinucleotide phosphate (NAADP), and cyclic ADP-ribose (cADPR), are believed to play a major role in the initiation of local Ca^2+^ signals by promoting Ca^2+^ release from internal Ca^2+^ stores. Enzymes generating these second messengers, such as phospholipase C (PLC) forming IP_3_ or the NAD glycohydrolase/ADP-ribosyl cyclase CD38 [forming cADPR in type 3 orientation with its active site facing inside the cytosol (9)], are localized in the plasma membrane. Thus, the second messengers must diffuse from sub-plasmalemmal space to their respective target channels. However, published diffusion coefficients of second messengers, such as IP_3_, may be overestimated (10). In 1992, an IP_3_ diffusion coefficient of 283 µm^2^ s^−1^ was determined in cytoplasmic oocyte extracts (11). Now, a 30-fold lower IP_3_ diffusion coefficient of ≤10 μm^2^ s^−1^ was calculated upon analysis of Ca^2+^ puffs evoked by IP_3_ photorelease in neuroblastoma cells. The latter corresponds to a more physiologic environment than cytoplasmic oocyte extracts. Accordingly, the range of action of IP_3_ is <5 µm (in contrast to 25 µm in oocyte extracts), indicating that IP_3_ may not spread throughout a large cell (10). However, due to the small size of T-lymphocytes, between 5 and 6 µm diameter for naïve cells and 9 and 12 µm for T cell blasts, IP_3_ may diffuse throughout the cytosol in naïve T cells. It is, however, unclear to what extend solid cell structures, such as organelles or the cytoskeleton may constitute diffusion barriers. Activity and localization of second messenger degrading enzymes play a further massive role in the second messengers’ spatiotemporal distribution, thereby directly influencing formation of Ca^2+^ microdomains.
  Up to now, NAADP is the most promising candidate for the generation of very early localized Ca^2+^ signals, which then trigger global Ca^2+^ signaling: NAADP is rapidly formed and reaches its peak concentration at (or also possibly below) 10 s post-activation (12, 13). NAADP is the most potent Ca^2+^-mobilizing second messenger known today, since it exerts already the highest Ca^2+^ signals at low nanomolar concentrations (12) (Figure 1). Though NAADP’s formation and molecular targets are still a matter of debate (12), there is growing evidence that NAADP acts *via* a specific binding protein (14–16).
  In T cells, further Ca^2+^-mobilizing second messengers, such as cADPR and IP_3_ are formed at later time points, in the minute range or over tens of minutes, and have been associated with a sustained cell activation, targeting the endoplasmic ryanodine receptor types 2 and 3 (RyR2, RyR3) and IP_3_R (17). Interestingly, glycerinaldehyde-3-phosphate dehydrogenase was very recently proposed as novel binding protein for cADPR (18). Activation of RyR and IP_3_R results in depletion of the ER, activating store-operated Ca^2+^ entry (SOCE) *via* activation of Stim1, which couples to the plasma membrane channel Orai1 (Figure 1).
(b) Ca^2+^ channels: Ca^2+^ channels involved in Ca^2+^ microdomain formation may either be located in the plasma membrane or in membranes of Ca^2+^ stores. The activation mechanisms of these channel families are very different; they range from physical stimuli, such as membrane depolarization or temperature changes, *via* activation by store depletion to activation by small molecular ligands. While the first two possibilities are often realized for Ca^2+^ channels located in the plasma membrane, activation by small molecular ligands is a hallmark of Ca^2+^ channels located in the ER (or SR) membrane. For T cells, membrane depolarization does not play a major role and will not be discussed here. Temperature changes may indeed play a role at sites of inflammation and induction of pyrexia; however, involvement of temperature-sensitive channels has not been described for T cells. The major players for T cells are plasma membrane Ca^2+^ channels activated by store depletion, such as Orai1, and ER channels, such as RyR1 and RyR3 [RyR2 apparently plays a minor role in effector T cells (19)] and IP_3_Rs. Involvement of Ca^2+^ release channels located on lysosomes, such as transient receptor potential (TRP) channels and two-pore channels (TPCs) have also been suggested [e.g., reviewed in Ref (12).]. In particular, TRPML1 and TRPM2 as well as TPC1/2 have been proposed as NAADP-sensitive channels; however, present data indicate that particularly ER Ca^2+^ contributes to early Ca^2+^ microdomains as will be discussed in Section “Characterization of Ca^2+^ Microdomains in T-Lymphocytes and Other Immune Cells.”
  In case of Orai1, the canonical view is that activation strongly depends on Ca^2+^ store depletion. However, in smooth muscle cells, growth factors activated Orai1 in the absence of Orai1/Stim1 cluster formation (20). Preformed clusters of IP_3_R have been suggested in non-lymphocyte mammalian cell types since single functional IP_3_R requires many seconds rather than milliseconds to diffuse within the ER membrane (21). These data indicate that initial Ca^2+^ microdomains occur due to preformed protein structures. Putative targets and microclusters in T-lymphocytes will be discussed in Section “Characterization of Ca^2+^ Microdomains in T-Lymphocytes and Other Immune Cells.”
(c) Ca^2+^ pump activation and recruitment: Plasma membrane calcium ATPase (PMCA) is activated by Ca^2+^ microdomains occurring near Ca^2+^ release-activated Ca^2+^ channels (CRAC) (22), thereby preventing intracellular Ca^2+^ overload and later restoring basal [Ca^2+^]_i_ levels. Interestingly, PMCA is activated already after 1 min of TCR activation in Jurkat T cells, reaching its steady state approximately 5 min post-activation (22). An accumulation of PMCA has been observed at the immune synapse, leading to a local decrease in Ca^2+^ concentrations, whereas global [Ca^2+^]_i_ remains increased in mathematical modeling of Ca^2+^ signaling in the immune synapse (23, 24). Maccari et al. (24) suggest that this mechanism may be part of a hierarchy preventing CRAC inactivation at the immune synapse: first, mitochondria serve as Ca^2+^ stores to guide incoming Ca^2+^ deeper into the cytosol. If mitochondria are not present, Ca^2+^ extrusion *via* PMCA accumulation diminishes local [Ca^2+^]. Furthermore, in the vicinity of the ER, sarco-endoplasmic reticulum calcium ATPase (SERCA) pumps Ca^2+^ back into the ER-lumen in order to reincrease ER Ca^2+^ content and to decrease [Ca^2+^]_i_. The increase of the luminal Ca^2+^ concentration then inactivates CRAC. Accordingly, the central role of PMCA and SERCA in the modulation of Ca^2+^ microdomain dynamics can be anticipated, though experimental data have not yet been published.
(d) Distribution of Ca^2+^ buffers: There are plenty of cytosolic and luminal Ca^2+^-binding proteins exerting Ca^2+^ buffering activity, thereby modulating spatiotemporal propagation of Ca^2+^ signals. In studies, modeling Ca^2+^ signaling in T-lymphocytes an intracellular concentration of Ca^2+^ buffers of 100 µM is presumed (25). Luminal Ca^2+^-binding proteins such as calnexin and calreticulin strongly accumulate in mitochondrial–ER junctions, thereby exerting regulatory effects, e.g., on SERCA, modulating ER and mitochondria Ca^2+^ storage and refilling (26). Furthermore, cytosolic Ca^2+^-binding proteins are, e.g., calmodulin, parvalbumin, and calbindin (27). However, expression levels of these proteins vary between cell types, and to the best of our knowledge, no data are available in leukocytes. The EF-hand-containing protein calmodulin plays a central role in T cell differentiation and proliferation since binding of Ca^2+^ induces calcineurin activation, which dephosphorylates NFAT enabling its nuclear translocation [reviewed in Ref (2).]. Ca^2+^ buffering is not only mediated by Ca^2+^-binding proteins but also mitochondria take up Ca^2+^ from microdomains (26). Due to the low affinity of the mitochondrial Ca^2+^ uniporter, mitochondrial Ca^2+^ concentration is closely associated with the occurrence of Ca^2+^ microdomains. Hence, Ca^2+^ microdomains are located in so called mitochondria-associated membranes, thus in close proximity to Ca^2+^ channels such as IP_3_R and RyR on the ER and CRAC in the plasma membrane, since Ca^2+^ microdomains are assumed to originate at these sites (26). Interestingly, in T cells where a mature synapse is not (yet) formed, occurrence of Ca^2+^ microdomains is increased, whereas in the established immune synapse, less Ca^2+^ microdomains and an increased global [Ca^2+^]_i_ were observed (23). This may (partly) depend on the before mentioned interplay of Ca^2+^ “sinks” PMCA, SERCA, and mitochondria. In T-lymphocytes particularly, uptake of Ca^2+^ into mitochondria accumulating at the immune synapse may lead to a sustained SOCE (26). As such, a rather “short” pulse of second messengers, such as IP_3_ which decreases 10 min after cell activation, is sufficient for a prolonged global Ca^2+^ signal since CRAC inactivation is inhibited, e.g., by mitochondrial Ca^2+^ uptake instead of SERCA-mediated ER refilling (28). Furthermore, mitochondrial Ca^2+^ uptake thereby prevents cellular Ca^2+^ depletion (28). In order to prevent a Ca^2+^-dependent inactivation of Orai1 channels, e.g., as shown in RBL-1 mast cells, mitochondria may accumulate at immune synapse and interact with protein components of the CRAC current, e.g., *via* mitofusin-2 (29, 30). Accordingly, accumulation of mitochondria has been inversely correlated with local Ca^2+^ signals in T lymphocytes in the immune synapse (23).

However, Ca^2+^ microdomains, generated by opening of one single channel, e.g., RyR or IP_3_R, may raise local Ca^2+^ rapidly into the dimension of 20–200 µM due to the fact that the Ca^2+^ flow increases too fast to be buffered (31). If the open probability of such a channel is continuously high, presumably, the Ca^2+^ diffusion coefficient increases approximately fivefold due to a saturation of the cytoplasmic buffering capacity (11).

## Function of Ca^2+^ Microdomains in T-Lymphocytes

Since in T cells, the endogenous NAADP concentration increases upon TCR/CD3 stimulation within seconds, there is good evidence that this Ca^2+^-mobilizing second messenger plays a major role in Ca^2+^ microdomain formation. This is also evidenced by recent data showing that knockdown of the three isoforms of RyR (RyR1–3) in T cells largely abolished NAADP-evoked Ca^2+^ microdomains (32). Since in T cells further Ca^2+^-mobilizing second messengers, such as IP_3_, are formed, the physiological role of the initial NAADP-dependent Ca^2+^ microdomain may be questioned. However, Ca^2+^ microdomains initiated by NAADP in T-lymphocytes are of high physiological relevance since they directly determine the amplitude of the subsequent global Ca^2+^ signal (19). Furthermore, in the multiple sclerosis animal disease model, experimental autoimmune encephalomyelitis (EAE), it was shown that antagonism of NAADP signaling with the pharmacological inhibitor BZ194, less autoreactive T-lymphocytes accumulated in the CNS and an alleviated clinical course of the EAE was observed (33).

Accordingly, understanding the mechanisms underlying formation of Ca^2+^ microdomains in T lymphocytes will give important insights into spatiotemporal aspects of fast signaling processes in non-excitable cells and further may open up avenues for novel targets to be used in T cell modulation. Regarding the underlying mechanisms, there are several ideas, possibly all of which may apply:

(a) Ca^2+^ microdomains are essential for the induction of a sustained, global Ca^2+^ increase *via* activation of further channels.
  One of the main hypotheses emphasizing the biological relevance of Ca^2+^ microdomains is that spatially restricted Ca^2+^ release in a trigger zone initiates and modulates the global Ca^2+^ increase. This was suggested by the so called two pool model: initial Ca^2+^ microdomains evoked by NAADP *via* RyR are then amplified by Ca^2+^-induced Ca^2+^ release (CICR) *via* RyR and IP_3_R (34). Such a model is supported by data from T cells showing NAADP-evoked Ca^2+^ microdomains and subsequent increase of global [Ca^2+^]_i_, dependent on RyR1 expression (19, 32, 33). Regarding the amplification channels, it is interesting to note that full opening of IP_3_R and RyR depends on the presence of the co-agonist, free Ca^2+^, besides binding of the respective second messengers IP_3_ and cADPR (17, 35, 36). Further amplification of T cell Ca^2+^ signaling is then realized by ER Ca^2+^ depletion, resulting in Ca^2+^ entry mediated *via* SOCE (Stim1/Orai1) (16, 34) (Figure 1).
(b) In a different model, initial Ca^2+^ microdomains in T-lymphocytes may not only result from NAADP activity but also may induce NAADP (and/or other second messenger) formation. This has been previously observed in germ cells (37, 38). NAADP will in turn bind *via* an accessory protein (14, 39) to its target receptors initiating secondary (not initial!) Ca^2+^ microdomains. These are then amplified by CICR and other second messengers, such as IP_3_ and cADPR binding their respective receptors.
(c) Localized signal transduction enables immune synapse formation. A mature immune synapse is formed within 2–60 min after antigen binding of the TCR. It is dependent on early Ca^2+^ signals leading to actin and signaling protein reassembly in the synapse (40, 41). The function of localized Ca^2+^ signals has not been clearly identified: opening of further Ca^2+^ releasing channels, e.g., IP_3_R, or enzyme activation within the immune synapse may be enabled (40). In 5C.C7 T-lymphocytes stimulated on a glass surface, Ca^2+^ flux has been observed within 6–7 s, while phosphorylation of LAT (linker for activation of T cells), a transmembrane protein centrally involved in propagation of TCR signaling upon Zap70 phosphorylation and formation of diacylglycerol were observed as faster events within 4 s. PhosphoLAT then induces cytoskeletal changes: reorientation of microtubule-organizing center, followed by visible alterations of tubulin reorientation within 20 s (42).
(d) For cytotoxic T-lymphocytes (CTLs), it was postulated that local, NAADP-dependent Ca^2+^ signals drive exocytosis of cytolytic granules (43). In CTL, other than in helper T cells (see above), NAADP may target TPCs on cytolytic granules thus forming Ca^2+^ microdomains leading to exocytosis. By contrast, a global [Ca^2+^]_i_ increase will only induce exocytosis if protein kinase C and the kinase ERK are activated (43). In CTLs, the release of lytic granules, cellular recognition, and apoptosis are initiated within the first 5 min of target cell recognition [reviewed in Ref (44).].
(e) Interestingly, Ca^2+^ microdomains generated by CRAC opening may differentially activate transcription factors as shown for c-fos and NFAT subtypes, which are both activated *via* Ca^2+^ signaling. Under regulation of caveolin-1, Ca^2+^ microdomains formed by CRAC opening may not only activate NFAT-mediated gene expression but also reduce c-fos-mediated transcription in RBL-1 mast cells (45). In a subsequent study, the group of Parekh found that NFAT1 is activated by Ca^2+^ microdomains, whereas NFAT4 is dependent on IP_3_R-mediated Ca^2+^ mobilization (shown in HEK293 cells) (46). These processes were observed 20–40 min post-activation (45, 46).

**Figure 2:**
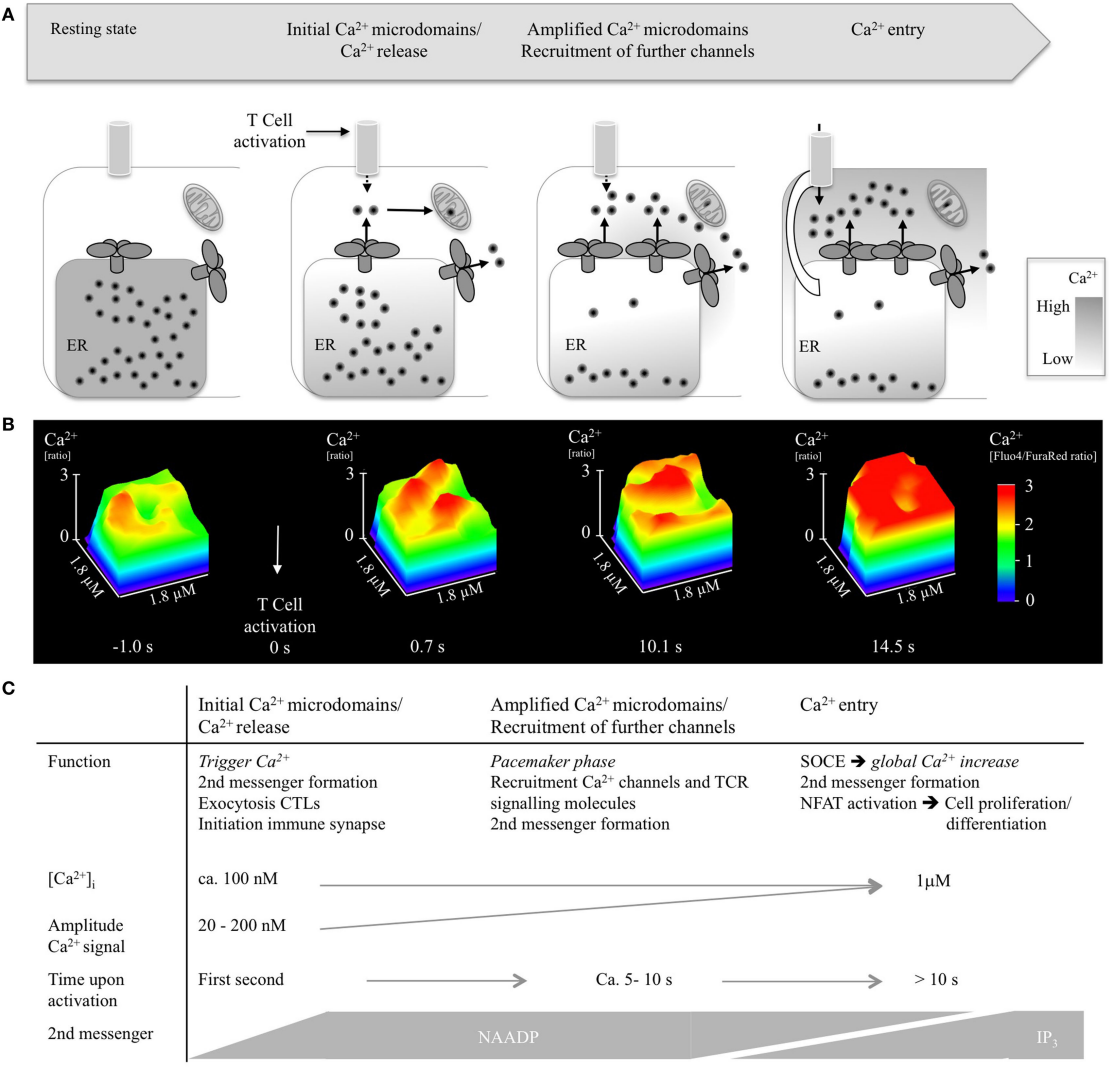
**Ca^2+^ microdomains in T-lymphocytes upon activation**. **(A)** Scheme indicating opening of single Ca^2+^ release channels, recruitment of further Ca^2+^ channels, and Ca^2+^ entry. **(B)** Progression of Ca^2+^ microdomains in a wild-type CD4^+^ T lymphocyte during the first 15 s of activation, shown as detail of a surface plot (1.8 µm× 1.8 µm). T-lymphocytes were isolated, loaded with cell permeable acetoxymethylesters of Fluo4 and FuraRed, activated using antibody-coated beads (anti-CD3/anti-CD28), imaged using a Leica IRBE2, combined with a Sutter DG4 and EMCCD Hamamatsu camera, and analyzed as previously reported (32). **(C)** Summarizing visualization of Ca^2+^ microdomain properties such as function, Ca^2+^ amplitude, time scale, and second messengers.

Taken together, the current picture of the mechanisms underlying initial Ca^2+^ microdomains consists of three phases: (i) initial Ca^2+^ microdomains occurring in the first second of T cell activation, (ii) recruitment of further channels within the next 5–10 s, and (iii) transition to global Ca^2+^ signaling by massive Ca^2+^ entry with tens of seconds (Figure 2). Phases (i) and (ii) depend on NAADP and its target receptor RyR1, but not on TRPM2 (TRP channel, melastatin 2) (29). Furthermore, an early Ca^2+^ entry pathway appears to be involved in phases (i) and (ii), too (29).

Propagation of Ca^2+^ microdomains in activated T-lymphocytes as well as characteristics and potential function of Ca^2+^ microdomains is delineated in Figure 2.

## Characterization of Ca^2+^ Microdomains in T-Lymphocytes and Other Immune Cells

T-lymphocyte Ca^2+^ microdomains were analyzed for the first time in 2003 (53). In Jurkat T-lymphocytes, Ca^2+^ signals close to the plasma membrane were characterized by an amplitude of 82–109 ± 30 nM with diameters between 2.5 ± 0.9 and 3.5 ± 1.5 μm and frequencies between 0.235 and 0.677 s^−1^. These signals were not significantly affected by blockade of Ca^2+^ entry or an Ca^2+^-free extracellular solution. Unfortunately, usage of the excitation shift Ca^2+^ indicator Fura-2 did not allow for sub-second analysis (sampling rate: 1 frame/1.5 s).

In Jurkat T cells with knockdown of RyR (likely all subtypes, but due to lack of subtype-specific antibodies not confirmed), Ca^2+^ microdomains close to the plasma membrane were decreased upon cell activation with soluble anti-CD3. Interestingly, upon cADPR microinjection and usage of a cell-permeable cADPR derivative (cIDPRE) in a RyR knockdown Jurkat clone, diminished Ca^2+^ microdomains were observed (12). Similar results were obtained upon pharmacological inhibition of RyR with ruthenium red. These data indicate that RyR are involved in Ca^2+^ microdomain formation in Jurkat T-lymphocytes (17). Furthermore, RyR are a target of cADPR. However, kinetics of endogenous cADPR upon TCR/CD3 stimulation does not support involvement of cADPR in Ca^2+^ microdomain formation (36).

Comparison of microinjection of NAADP, cADPR, or IP_3_ into Jurkat T-lymphocytes gave important insights into the mechanism underlying initial Ca^2+^ microdomains (54). Ca^2+^ microdomains of approximately 0.5 µm size were observed near the injection site for all three second messengers. Interestingly, NAADP-mediated signals were abolished upon co-injection of the RyR inhibitors ruthenium red and ryanodine, but not by co-injection of the IP_3_ inhibitor heparin or the Ca^2+^ entry blocker SKF 96365 (54). These data also strongly indicate that initial NAADP-dependent Ca^2+^ microdomains in T-lymphocytes are RyR dependent. Furthermore, in RyR knockdown cells, NAADP microinjection did not initiate localized nor global Ca^2+^ signals (54), as discussed above.

Using an improved measurement setup with an increased spatiotemporal resolution (sampling rate: 25 ms/frame), we were able to support these data in primary mouse RyR1^−/−^ T cells. RyR knockdown in Jurkat T lymphocytes as well as the RyR1 knockout in primary mouse CD3^+^ cells resulted in an inhibition of initial Ca^2+^ microdomains, leading to a delayed and diminished global Ca^2+^ signal. Within the first 130 ms upon TCR stimulation with anti-CD3 coated beads, localized Ca^2+^ microdomains occurred in Jurkat T lymphocytes very close to the plasma membrane (32). These microdomains are in close proximity to the activation site with peak Ca^2+^ concentrations exceeding 115 nM Ca^2+^ [similar to previous findings (53)] and a spatial spread close to the resolution limit.

In three different CTL cell lines, there is evidence that TPC on cytolytic granules may be the target of NAADP, initiating release of perforin and granzyme into the synaptic cleft (43, 55). Interestingly, TPC was found to accumulate in the immune synapse, which may allow for rapid release of cytolytic proteins (55). Furthermore, particularly local Ca^2+^ signals together with NAADP may be of central importance for initiation of exocytosis, while a global [Ca^2+^]_i_ increase requires protein kinase C and ERK activation. Interestingly, treatment with the Ca^2+^-ATPase inhibitor CPA resulted in an abrogation of Ca^2+^ signals, suggesting that ER Ca^2+^ signals are prerequisites (not contributors!) for initial Ca^2+^ signals also in CTL (55). Thus, in Jurkat T-lymphocytes, the target channel and organelle of NAADP remain unclear: while acidic stores were ruled out as NAADP target by Steen and co-workers (56), others found targeting of TPC on lysosomes by NAADP (43).

Similar to CTLs, Ca^2+^ microdomains were observed in neutrophils close to the interface of neutrophil–tumor cell synapses over approximately 40 min of measurement. In the interface, Stim1 was enriched, whereas Ca^2+^-binding proteins such as calbindin and parvalbumin were not (57). Furthermore, Stim1 has been shown to recruit ER cisternae near phagosomes. Ca^2+^ microdomains generated by periphagosomal ER and phagosomal Ca^2+^ stores may then promote efficient phagocytosis in neutrophils and are decreased by 50% in Stim1-depleted neutrophils (58). These data strongly support the importance of Ca^2+^ microdomains in the ER–plasma membrane interface and the interplay of Ca^2+^ entry and release mechanisms. However, no data on early Ca^2+^ microdomain formation were acquired in these studies (42).

Junek et al. investigated Ca^2+^ release from different compartments in Ramos and DT40 B-lymphocytes (49). In B-lymphocytes, the reassembly of a multiprotein complex comprising Src homology [SH2] domain-containing leukocyte protein of 65 kDa (SLP65), CIN85, BtK, and phospholipase C-γ2 resulted in (presumably IP_3_-mediated) Ca^2+^ release from the ER (approximately 8 s after SLP65 recruitment). Interestingly, the Ca^2+^ signal started to spread from the central region of the cell, and simultaneously [Ca^2+^] increased in mitochondria, suggesting Ca^2+^ buffering. In addition, [Ca^2+^] also increased in Golgi system, which may play a role in vesicle trafficking during later stages of Ca^2+^ signaling (49).

TRPV1 (TRP, vanilloid 1) contributes to TCR-induced Ca^2+^ influx in a tyrosine phosphorylation-dependent fashion. Though potentially interesting, TRPV1 effects were not analyzed on a fast time scale (59).

## Microdomain Organization in T-Lymphocyte Signaling

Several studies point to the importance of characteristic (non-Ca^2+^) microdomains involved in T cell signaling. With a focus on the initial 10 s upon T cell activation, the following processes are described in the literature.

T-lymphocytes respond to antigen presentation by its cognate MHC within seconds (42). Particularly preformed microclusters of TCR and other signaling molecules have been observed and are capable of rapidly initiating intracellular signaling processes (60–62). In Th1 and Th2 cells, different patterns in TCR microdomain organization have been observed. Upon activation, Th1 cells respond with a sustained Ca^2+^ signal and the plasma membrane is characterized by lipid rafts with microdomains rich in TCR and Kv channels (63). By contrast, Th2 cells only respond with a brief Ca^2+^ increase and the plasma membrane consists of less and smaller TCR-rich lipid rafts (63). Thus, non-Ca^2+^ microdomains clearly determine cell signaling and vary between the T cell subsets. Similarly, preformed clusters of Stim and Orai have been suggested which may rapidly initiate CRAC (64).

Analyzing the very first seconds of T cell activation, “tri-phasic” Ca^2+^ signaling has been observed in Jurkat and primary murine T-lymphocytes (as described in Section “Function of Ca^2+^ Microdomains in T-Lymphocytes”). Initial Ca^2+^ microdomains were observed immediately following activation, and decreased in intensity after the first second (32). These signals depend on RyR1.

**Figure 3:**
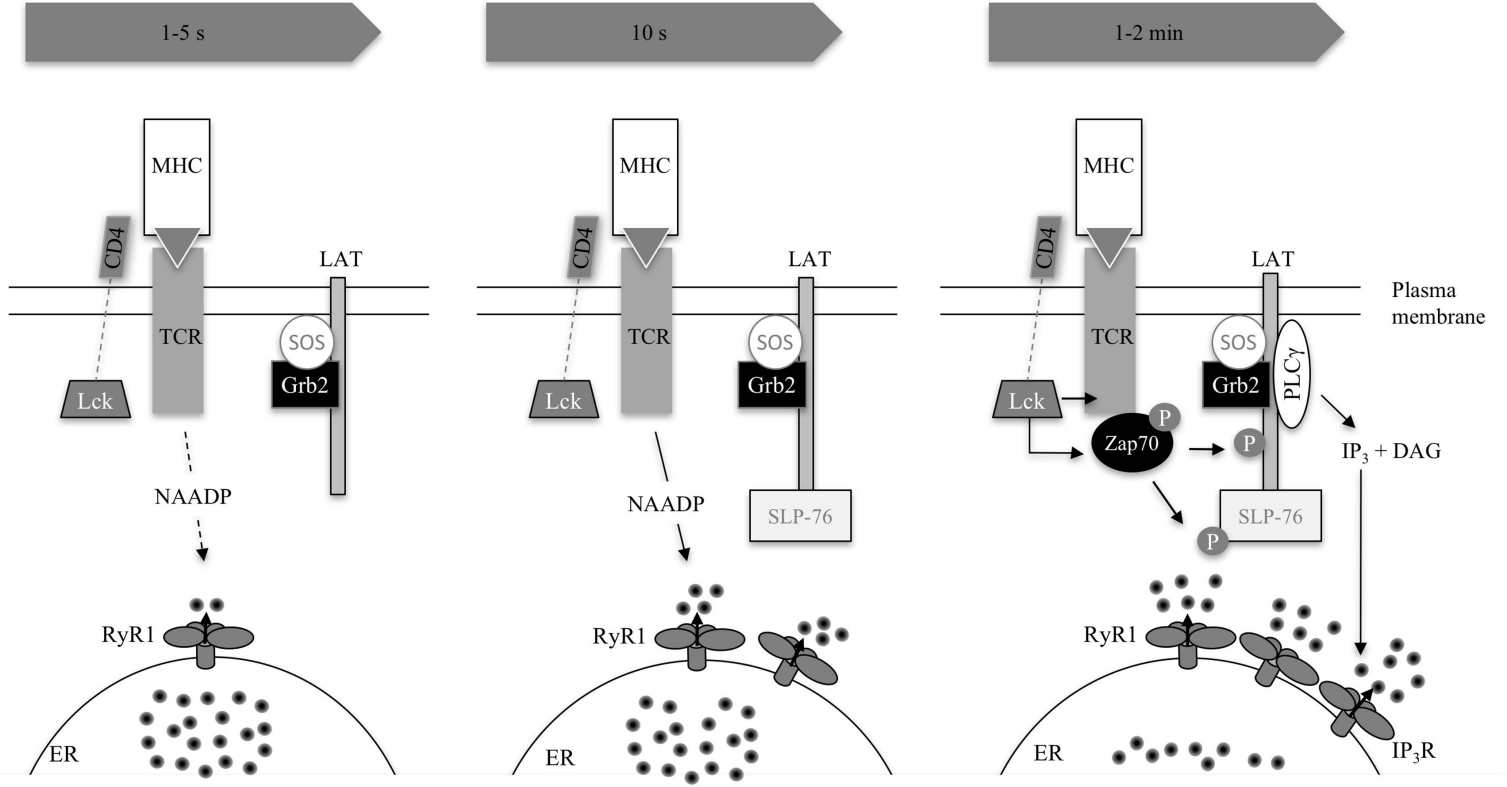
**Excerpt of TCR signaling in early Ca^2+^ release**. The three consecutive images condense early signaling 1–5 s, 10 s, and 1–2 min post-activation as summarized in Table 1. The TCR binds to its cognate antigen. In principle, this activates the SRC kinase LCK, which phosphorylates immunoreceptor tyrosine-based activation motifs (not shown) in the CD3 subunits of the TCR complex. These CD3 subunits recruit and activate Zap70, which phosphorylates LAT (1–2 min) and SLP76 (10 s). SLP76 is related to Src homology [SH2] domain-containing leukocyte protein of 65 kDa (SLP65) in B-lymphocytes but is not functionally identical (47). LAT recruits SLP76 to the cell membrane and a multimolecular complex is formed recruiting, e.g., PLCγ or the guanine exchange factor VAV (not shown). Interestingly, SLP65 has already been recruited 10 s upon activation. The adaptor protein Grb2 is constitutively bound to exchange factor son of sevenless (SOS). LAT recruits Grb2 and SOS thus activates the GTPase Ras, a crucial activator of MAPK pathways (48). The second messenger nicotinic acid adenine dinucleotide phosphate is formed by an unknown enzyme and activation process 10 s post-TCR activation. IP_3_ rises consecutively, formed by PLCγ. Both second messengers lead to Ca^2+^ release on their respective receptors RyR1 and IP_3_R located on the endoplasmic reticulum (ER) (40, 42, 47–52).

Interestingly, microdomains of Grb2, an adaptor protein of LAT (Figure 3), increased 4 s following photoactivation of an antigenic peptide, indicating that LAT phosphorylation follows initial Ca^2+^ microdomains (42). It is unclear which biological function may underlie this time delay, proofreading (whether a genuine agonist is bound), or time consumption for biochemical processes of signal transduction, e.g., second messenger formation (i.e., NAADP biosynthesis), are putative explanations (42).

Seven seconds after the initial Ca^2+^ microdomains, a second occurrence of Ca^2+^ microdomains was observed in Jurkat and primary murine T-lymphocytes (32). This is in accordance with previous data in 5C.C7 T cell blasts. Here, a Ca^2+^ signaling delay (“offset time”) of 6.5 ± 0.5 s following photorelease of an antigenic peptide presented by an antigen-presenting cell (APC) was observed (42). Possibly, the very initial Ca^2+^ microdomains in the first second were not detectable due to an overlap of the antigen-releasing UV flash. The global Ca^2+^ peak is reached approximately 10–20 s following cell activation (40, 49).

There are data indicating that LAT phosphorylation occurs mainly downstream of Ca^2+^ signals at around 10 s, which is in contrast to the findings of Huse et al. (42): Zap70, which phosphorylates LAT after having bound to the immunoreceptor tyrosine-based activation motifs of the antigen-binding TCR, accumulates steadily after contact with a superantigen-pulsed B-cell in Jurkat T lymphocytes over 2 min finally reaching its plateau, whereas a [Ca^2+^]_i_ peak occurs earlier at 10 s (41) (Figure 3). These data strongly support the idea that there must be a Zap70 phosphorylation-independent mechanism leading to early Ca^2+^ microdomains in T cells. Since phosphorylated LAT attracts PLCγ, activating the Ca^2+^ signaling cascade *via* IP_3_, these data are in accordance with the assumption that IP_3_ is formed with a time delay (Figure 2). It remains to be elucidated whether phosphorylation-dependent mechanisms play a role in early Ca^2+^ microdomain formation.

Table 1 lists in chronological sequence T cell activation events and the respective underlying processes, such as second messenger formation and protein recruitment.

**Table 1:** **Consolidated chronological sequence of local and global Ca^2+^ signaling, second messenger formation, and protein recruitment following immune cell activation**.

Time	Process	2nd messenger	Ca^2**+**^ release	Other	Cell type	Reference
0 s	Activation of TCR/BCR			Preformed TCR rich lipid rafts	mmTh1, Th2 cells	(63, 64)
				Preformed Stim/Orai		

1 s	Trigger Ca^2+^		RyR1		Jurkat T cells; mmCD3^+^	(32)

4 s				Grb2 microdomain formation	5C.C7 T cell blasts	(42)

5–10 s	Amplified trigger Ca^2+^	Nicotinic acid adenine dinucleotide phosphate (NAADP) (40 nM)	RyR1		Jurkat T cells; mmCD3	(32, 50)

10 s (8 s prior free cytosolic Ca^2+^ concentration onset)				Src homology [SH2] domain-containing leukocyte protein of 65 kDa translocation to plasma membrane	Ramos and DT40 B cells	(49)

10 s	Ca^2+^ peak				Jurkat T cells	(40)

20 s	Ca^2+^ peak				Ramos and DT40 B cells	(49)

20 s				Tubulin reorientation, cytoskeletal change	5C.C7 T cell blasts	(42)

30 s		Cyclic ADP-ribose (cADPR)			Killer cells (LAK)	(65)

1 min				Activation plasma membrane calcium ATPase (PMCA)	Jurkat T cells	(22)

1.5 min		NAADP			Killer cells (LAK)	(65)

2 min				Peak Zap70 accumulation	Jurkat T cells	(40)

3 min		IP_3_	IP_3_R		Jurkat T cells	(51)

5 min	Global Ca^2+^			PMCA steady state	Jurkat T cells	(22)

5–10 min		NAADP (20 nM)			Jurkat T cells	(50)

4–8 min				Calcineurin → nuclear factor of activated T cells; AP1; NF-κB	B cells	(66)

10 min		cADPR	RyR3			(17, 36)

NN				Store-operated Ca^2+^ entry *via* Ca^2+^ release-activated Ca^2+^ channels		(44)

NN				Enrichment of Kv1.3 in IS		(67)

NN				Accumulation of mitochondria in IS		(23, 68)

NN		NAADP		Exocytosis	Cytotoxic T-lymphocytes	(43)

*IS, immune synapse; NN, without kinetic/time point; mm, mus musculus*.

## Conclusion

Data analyzing localized Ca^2+^ events on a fast time scale and in the first seconds of stimulation are rather scarce in T-lymphocytes. Not only that detection of futile Ca^2+^ signals in a surrounding with plenty of Ca^2+^-binding proteins and organelles is difficult *per se*, T-lymphocytes are more difficult to analyze due to their spherical cell shape. However, there are promising tools and methods facilitating measurement of Ca^2+^ microdomains, e.g., the combination of Ca^2+^ indicators, such as FuraRed and Fluo4, to attain ratiometric data without time shift or novel nanobiosensors (32, 69, 70). The latter, cell permeable nanobiosensors, enable the pointillistic readout of Ca^2+^ signals using TIRFM applications. Furthermore, fiber-based nanobiosensors were developed to track intracellular Ca^2+^ microdomains (8). However, only few data with these new techniques are available and, to our knowledge, have not yet been used in T-lymphocytes (69). The functional analysis of Ca^2+^ microdomains further necessitates the directed stimulation of T-lymphocytes. Thus, either coated surfaces (42), APCs such as B-lymphocytes, or antibody-coupled beads may be used to simulate TCR activation in an immune synapse (71–73).

Major pitfalls of microdomain measurement in general are rather slow diffusion and reaction time of biosensors (41). For example, Src and supposedly Zap70 sensors show a relatively slow estimated distribution of 0.93 ± 0.06 µm^2^/s within the cytoplasm and even slower in the membrane (at lipid raft: 0.11 ± 0.01 μm^2^/s and outside 0.18 ± 0.02 μm^2^/s) (74). Furthermore, indicator photobleaching determines sampling rate, leading to a limitation in sampling: in Zap70 experiments, carried out at 5 s measurement intervals, photobleaching predominates diffusion of indicator thereby disabling quantitative measurements (41). Accordingly, data on initial phosphorylation processes induced by TCR-activation are still not precise enough to describe in detail kinetics of intracellular signal transduction.

Table 1 summarizes the current data on the chronological sequence of (early) Ca^2+^ signal formation. Present data suggest that NAADP is the driving second messenger in early Ca^2+^ microdomain formation in T-lymphocytes and other immune cells. Possibly, NAADP formation or its binding to an unidentified accessory binding protein may be dependent on initial Ca^2+^ signals occurring in the first second of TCR activation. Interestingly, the NAADP target receptor RyR1 gives rise to initial Ca^2+^ microdomains (first 10 s of cell activation). By contrast, TPC apparently contribute to Ca^2+^ microdomain signaling in CTL, however, spatiotemporal data for TPC activation in primary CD4^+^ T-lymphocytes are still missing. To date, there is no indication that other channels such as TRPM2 or TRPV1 are involved in early (NAADP-mediated) Ca^2+^ microdomain formation by Ca^2+^ release from intracellular stores. Mitochondria apparently may play a central role in buffering Ca^2+^ microdomains in the immune synapse to prevent inactivation of Orai1.

Taken together, presently Ca^2+^ signaling events can be determined with high spatiotemporal resolution, while for other well-known T cell activation events, such as tyrosine phosphorylation, advanced methods are required. Nevertheless, the chronological sequence presented here may stimulate new approaches to understand the interactions between the different signaling modules involved in T cell activation.

## Author Contributions

Both authors (IW and AG) wrote and approved the manuscript.

## Conflict of Interest Statement

The authors declare that the research was conducted in the absence of any commercial or financial relationships that could be construed as a potential conflict of interest.

## Abbreviations

APC, antigen-presenting cell; [Ca^2+^]_i_, free cytosolic Ca^2+^ concentration; cADPR, cyclic ADP-ribose; CICR, Ca^2+^-induced Ca^2+^ release; CRAC, Ca^2+^ release-activated Ca^2+^ channels; CTLs, cytotoxic T-lymphocytes; IP_3_, d-*myo*-inositol 1,4,5-trisphosphate; ITAM, immunoreceptor tyrosine-based activation motif; ER, endoplasmic reticulum; LAT, linker for activation of T cells; MCU, mitochondrial Ca^2+^ uniporter; NAADP, nicotinic acid adenine dinucleotide phosphate; NFAT, nuclear factor of activated T cells; PLC, phospholipase C; PMCA, plasma membrane calcium ATPase; sER; smooth ER; SERCA; sarco-endoplasmic reticulum calcium ATPase; SLP65, Src homology [SH2] domain-containing leukocyte protein of 65 kDa; SOCE, store-operated Ca^2+^ entry; TPC, two-pore channel; TRP, transient receptor potential.
